# Impact of hypothalamic reactive oxygen species in the regulation of energy metabolism and food intake

**DOI:** 10.3389/fnins.2015.00056

**Published:** 2015-02-24

**Authors:** Anne Drougard, Audren Fournel, Philippe Valet, Claude Knauf

**Affiliations:** NeuroMicrobiota, European Associated Laboratory, INSERM/UCL, Institut National de la Santé et de la Recherche Médicale, U1048, Institut des Maladies Métaboliques et Cardiovasculaires (I2MC), CHU Rangueil, Université Paul SabatierToulouse, France

**Keywords:** ROS, hypothalamus, food intake, metabolism, diabetes, obesity

## Abstract

Hypothalamus is a key area involved in the control of metabolism and food intake via the integrations of numerous signals (hormones, neurotransmitters, metabolites) from various origins. These factors modify hypothalamic neurons activity and generate adequate molecular and behavioral responses to control energy balance. In this complex integrative system, a new concept has been developed in recent years, that includes reactive oxygen species (ROS) as a critical player in energy balance. ROS are known to act in many signaling pathways in different peripheral organs, but also in hypothalamus where they regulate food intake and metabolism by acting on different types of neurons, including proopiomelanocortin (POMC) and agouti-related protein (AgRP)/neuropeptide Y (NPY) neurons. Hypothalamic ROS release is under the influence of different factors such as pancreatic and gut hormones, adipokines (leptin, apelin,…), neurotransmitters and nutrients (glucose, lipids,…). The sources of ROS production are multiple including NADPH oxidase, but also the mitochondria which is considered as the main ROS producer in the brain. ROS are considered as signaling molecules, but conversely impairment of this neuronal signaling ROS pathway contributes to alterations of autonomic nervous system and neuroendocrine function, leading to metabolic diseases such as obesity and type 2 diabetes. In this review we focus our attention on factors that are able to modulate hypothalamic ROS release in order to control food intake and energy metabolism, and whose deregulations could participate to the development of pathological conditions. This novel insight reveals an original mechanism in the hypothalamus that controls energy balance and identify hypothalamic ROS signaling as a potential therapeutic strategy to treat metabolic disorders.

## Introduction

The role of the brain in the control of energy homeostasis has been suggested at the middle of the nineteenth century by Claude Bernard who was the first to evoke its importance with the famous experiment “la piqûre diabétique” (Bernard, 1849). Indeed, he observed an important rise of glycemia after a stimulation of the floor of the fourth ventricle. In 1953, Gordon Kennedy identified the precise brain area involved in this phenomenon. He has demonstrated that circulating factors released by the adipose tissue could reach the hypothalamus in order to modulate food intake and energy expenditure, and thus regulate body weight (Kennedy, 1953). Moreover, the medio-basal hypothalamus is a privileged central area which directly senses various factors (hormones, metabolites). In fact, at the close proximity of the arcuate nucleus (ARC), the median eminence is a circumventricular organ that presents a blood-brain barrier-free area that allows peripheral factors to inform hypothalamic structures about the energy status (Rodriguez et al., 2010). Since 1940, the development of stereotaxic methods in animals has offered researchers the opportunity to practice stimulation or localized destructions of various hypothalamic nuclei, and then identified their precise role (Brobeck, 1946).

The first neurons in contact with peripheral signals are called “neurons of first order.” These neurons are only present in the ARC (Cone et al., 2001) and a lesion of this hypothalamic region induces hyperphagia and obesity (Olney, 1969). It is largely well described in the literature that the ARC presents two subsets of neurons that have opposite effects on feeding. One of these group of neurons express pro-opiomelanocortin (POMC) and cocaine-amphetamine-regulated transcript (CART), and upon stimulation, these neurons produce anorectic effects (Cone, 2005). The POMC precursor is cleaved into α-melanocyte-stimulating hormones (α-MSH) which reduces food intake, body weight and increases energy expenditure (Kim et al., 2000; Yasuda et al., 2004). The second group of neurons in the ARC leads to orexigenic responses upon activation. These neurons express neuropeptide Y (NPY) and the agouti-gene-related transcript (AgRP) (Clark et al., 1984; Rossi et al., 1998) which stimulate food intake and reduce energy expenditure (Fekete et al., 2002). In genetically-modified obese rodents, as well as in fasted normal animals, NPY mRNA expression and protein content are elevated in ARC (Sanacora et al., 1990; Wilding et al., 1993; Park et al., 2004). Collectively, this suggests that (1) over-activation or dysfunction of NPY neurons is involved in the emergence of metabolic diseases, but also that (2) NPY neurons can inhibit melanocortin system. Interestingly, subsets of POMC neurons contain both gamma amino butyric acid (GABA) and glutamate (Dicken et al., 2012). Subsets of NPY neurons also contain GABA (Horvath et al., 1997) and GABA from NPY neurons participates to inhibition of POMC neurons. This unidirectional input from the NPY to POMC cells brings to light one anatomical basis of energy regulation. This neuronal interaction seems to favor the tonic inhibition of satiety signal, and also promotes feeding or over-feeding when food is available in excess. This finding reinforces the key role of an intact melanocortin system in the control of energy balance.

The POMC and NPY/AgRP neurons of the ARC interact with various brain regions that include hypothalamic areas such as ventromedial hypothalamus (VMH), dorsomedial hypothalamus (DMH), paraventricular nucleus (PVN) and lateral hypothalamus (LH) (Wynne et al., 2005). Lesion studies in rodent have shown that ablation of VMH, DMH or PVN results in hyperphagia and obesity (Leibowitz et al., 1981; Shimizu et al., 1987; Bellinger and Bernardis, 2002) whereas lesions in the LH lead to hypophagia (Bernardis and Bellinger, 1993). To summarize, a proposal of dual-center model has been proposed that identifies the VMH as the “satiety center” and the LH as the “hunger center” (Stellar, 1994).

These hypothalamic nuclei have the ability to sense the level of available fuel energy in the body. This detection of peripheral signals involves various molecular actors such as classical neurotransmitters but also “non-classical” diffusible molecules such as reactive nitrogen species (RNS) and reactive oxygen species (ROS). To this aim, nitric oxide (NO) is an atypical molecular actor which belongs to RNS family, but could also be associated to ROS family since it is a radical gas derived from nitric and oxygen. NO has a very short half-life and can largely diffuse in cells (Palmer et al., 1987). It has a single electron, allowing numerous interactions with cellular components. In the brain, chemical properties of NO permits its diffusion across hypothalamic cells to control neurotransmitter release (Ohkuma and Katsura, 2001), but also synaptic plasticity (Shibuki and Okada, 1991), that leads to the modulation of food intake (Morley and Flood, 1991) and glucose metabolism (Duparc et al., 2011a). In addition to RNS, recent data argue that differential fuel utilization in response to nutrients is linked to ROS formation. This ROS generation is not merely a “byproduct” of substrate oxidation, but has a crucial role in regulating neuronal response in a substrate-dependent manner.

In this review, we will describe the molecular mechanisms generating ROS in response to metabolic signals, in order to control metabolism and food intake. In physiological conditions, ROS production is considered as a signaling actor produced at low level and in a transient manner. On the other hand, over-production of ROS, associated to a dysfunction of ROS buffering systems, can lead to the development of chronic diseases. Thus, central chronic ROS release participates in the establishment of numerous diseases such as type 2 diabetes (T2D), but also in cancer and neurodegenerative disorders (as Parkinson and Alzheimer disease).

## ROS production: focus on neuronal cells

### Introduction

ROS are radical species derived from oxygen of which the most known are the superoxide anion (O^−^_2_), the hydrogen peroxide (H_2_O_2_) and the hydroxyl radical (OH^−^). These “free-radical” ROS have unpaired electrons on an oxygen atom. This property makes ROS very unstable as they react quickly with others surrounding chemical molecules in order to pair their electrons. These radical reactions often lead to a new radical formation generating a chain reaction, and thus activate numerous signaling pathways (Gutteridge and Halliwell, 2000).

Within the cell, all processes using oxygen may produce ROS. However, the mitochondrial transport electron chain is a major source of ROS generation in physiological conditions (Bashan et al., 2009). ROS are also produced by the NADPH oxidases (NOX) which are membrane-bound enzymatic complexes able to transfer electrons from cytosolic NADPH to oxygen (Lambeth, 2004; Hordijk, 2006). Finally, another source of ROS is the endoplasmic reticulum (ER) (Cross and Jones, 1991) and the peroxisome (Schrader and Fahimi, 2006).

In order to limit ROS concentration, cells are equipped with various enzymatic and non-enzymatic antioxidant systems. Several enzymes directly degrade ROS such as the superoxide dismutase (SOD), the catalase and the glutathione peroxidase (Turrens, 2003). Various non-enzymatic scavengers can also support detoxification of free radicals. The majority are lipophilic (vitamin E, ubiquinone or coenzyme Q, and carotenoids) or hydrophilic (vitamin C, glutathione) (Clark, 2002).

When ROS are moderately and transiently produced, they are able to reversibly modify some surrounding targets and act as “signal” molecules (Droge, 2002). However, when oxidants-antioxidants balance is altered, due to an excessive production of ROS and/or a decrease in antioxidant systems, and is associated to the expansion of oxidative stress (Droge, 2002), ROS become toxic to the cell, and could be involved in the development of specific diseases markers such as insulin resistance and type 2 diabetes (Bashan et al., 2009).

### ROS and neurons

ROS are also strongly present in the brain. The large majority of ROS produced by neurons have a mitochondrial origin (Bao et al., 2009). However, localization of NOX in different regions of the central nervous system have been determined by immunohistochemical approaches in rats (Kim et al., 2005) and mice (Serrano et al., 2003). Indeed, NOX are present in hippocampus, cortex, amygdala, striatum, thalamus and hypothalamus.

ROS can act directly on the brain to control many central functions. Neurons can sense, transmit and convert ROS signals into appropriate intracellular responses, including synaptic plasticity. For example, NOX activity in the hippocampus is required to induce normal cellular process in these neurons, such as long-term potentiation (a form of activity-dependent synaptic plasticity) and hippocampus-dependent memory (Infanger et al., 2006). ROS production by mitochondria also occurs in the hypothalamus, and NOX subunits are also present in the ARC, VMN, and PVN (Infanger et al., 2006). Hypothalamus is a key area involved in control of glucose homeostasis (Knauf et al., 2013). Indeed, a population of specialized hypothalamic neurons is identified as gluco-sensors (Oomura et al., 1964). These neurons are classified into two categories, which differ both by their mode of action and by their precise localization, i.e., neurons excited by glucose (named glucose-excited or GE) and neurons inhibited by glucose (named glucose-inhibited or GI). Raising plasma glucose level after a large meal results in an increased brain level to 4,5 mM and an increase in brain glucose activates GE neurons, while GI remain inactive (Routh, 2002). At the opposite, during a period of fasting, reduction in the concentration of brain glucose inhibits GE neurons and activates GI ones (Routh, 2010). Although K_ATP_ channels seem to be essential for the function of gluco-sensors, several studies show a depolarization of these neurons via K_ATP_ channels independent mechanisms (Ainscow et al., 2002; Fioramonti et al., 2004). Among these mechanisms, mitochondrial ROS (mROS) are clearly involved in hypothalamic glucose sensing (Leloup et al., 2006). Indeed, a transient increase of glucose stimulates mROS generation on hypothalamic slices *ex vivo*. In addition, intra-carotid glucose load (therefore only detectable by the hypothalamus) in rats triggers a significant increase of ARC neuronal activity (Leloup et al., 2006) which is abolished by a co-injection of the antioxidant enzyme catalase. These results demonstrate that hypothalamic glucose sensing requires the acute generation of ROS as signaling molecules to induce a neuronal response.

Strong evidences demonstrate that ROS action in the brain can affect most of the peripheral tissues via the Autonomous Nervous System (ANS). For example, Zimmerman et al., observed that O^−^_2_ was necessary to elicit vasopressor and bradycardiac responses produced by intracerebroventricular (icv) administration of Angiotensin-II (Ang-II) in mice. Indeed, scavenging O^−^_2_ in brain completely abolished cardiovascular actions of Ang-II (Zimmerman et al., 2002). Moreover, central administration of a SOD mimetic attenuates the increase in renal sympathetic nerve activity, norepinephrine release and blood pressure induced by icv injection of Ang-II (Campese et al., 2004, 2005; Lu et al., 2004). Therefore, ROS in the brain are of crucial importance to provide signals in periphery via autonomous nervous system.

Thus, ROS have a strong role as signaling molecules in the central nervous system, and more particularly in the hypothalamus. Since hypothalamus is a key area involved in the control of energy homeostasis, we are now going to focus on the role of hypothalamic ROS in the regulation of energy metabolism and food intake.

## Involvement of hypothalamic ROS in the control of metabolism and food intake

We have previously discussed that a physiological increase of ROS levels has a signaling role in the hypothalamus. This brain area receives signals from the periphery such as nutrients (glucose and lipids) and hormones, which in turn are able to modulate the activity of hypothalamic neurons. This effect on hypothalamic neurons, including POMC and NPY/AgRP neurons, further impact peripheral fuel metabolism via the ANS or neuroendocrine mediators. Here, we will describe the effect of nutrient action (such as glucose and lipids) and hormones on hypothalamic ROS release, associated with peripheral metabolic regulation and food intake.

### Nutrients

Glucose and lipids are directly detected by hypothalamic neurons in order to implement the adaptive response for the metabolism. An intracarotid injection of glucose, which targets specifically the hypothalamus, generates a transient release of hypothalamic ROS (Leloup et al., 2006). This release of hypothalamic ROS induces, via the parasympathetic nervous system outflow, a peak of insulin 1–3 min later with no change in peripheral blood glucose. Scavenging ROS generation in the brain by the use of antioxidant molecules significantly disturbs insulin secretion induced by the cerebral glucose load, demonstrating the key role of hypothalamic ROS in the control of energy metabolism. In this experimental model, ROS production involved in glucose sensing has a mitochondrial origin. In fact, glucose co-injected with the mitochondrial uncoupler CCCP decreases significantly the peak of insulin (Leloup et al., 2006). If we focus on food intake, a VMH glucose infusion attenuates the increase of food intake after refeeding following an overnight fast, via a mechanism dependent on hypothalamic mROS production (Carneiro et al., 2012).

Concerning lipids, the literature also showed that these circulating nutrients can directly act as signaling molecules in the hypothalamus (Lam et al., 2005). A provoked acute hypertriglyceridemia in rats generates an acute ROS production in the VMH 30 min after an intraperitoneal injection of lipids (Benani et al., 2007). This hypothalamic ROS release is local, rapid and transient since it returns to basal levels within 4 h after injection. Therefore, this hypothalamic ROS modulation is able to inhibit food intake only 1 h after lipid injection. This anorectic effect is directly attributable to hypothalamic ROS release since icv infusion of antioxidant drugs, such as glutathion and trolox (an analog of vitamin E), abolishes the decrease of food intake. Similarly to glucose, lipids generate hypothalamic mitochondrial ROS production concomitant to an increase in mitochondrial respiration (Benani et al., 2007).

We described above that glucose and lipids generate hypothalamic ROS release through an action on mitochondria. Mitochondrial morphology is associated with mROS production and energy status (Yoon et al., 2011). Mitochondria are dynamic organelles that continuously change their structures under the control of different sets of proteins through fission and fusion mechanisms. A recent study has demonstrated that, in AgRP neurons, changes of mitochondrial morphology (size and number) are associated with the metabolic state of these neurons (Dietrich et al., 2013). Indeed, food deprivation during 24 h leads to an increase in mitochondria density and a decrease in mitochondria size in AgRP neurons, which are characteristic of changes in mitochondria dynamic toward fission, while neighboring anorexic POMC neurons display a decrease in mitochondria density. Conversely, high-fat-diet feeding (HFD) (i.e., positive energy balance) leads to mitochondrial fusion in AgRP neurons. These changes were cell-type specific because this phenomenon did not occur in POMC neurons. The fission machinery involves dynamin-related protein 1 (DRP1), which is translocated to the mitochondrial membrane where it forms a contractile “ring” involved in the division of mitochondria. Under basal conditions, DRP1 is cytoplasmic. In response to an intracarotid glucose bolus injection, DRP1 level is increased in mitochondria from VMH extract 1 min post-injection, and correlates with hypothalamic mROS production (Carneiro et al., 2012). These results indicate that mitochondrial fission is necessary for the induction of hypothalamic mROS release in response to glucose. Inhibition of DRP1 recruitment in the VMH mitochondria using siRNA is deleterious by the loss of hypothalamic mROS signaling, insulin release and the satiating effect of glucose. On the other hand, fusion machinery involves mitofusins (Mfn 1 and Mfn2). AgRP-specific Mfn1 or Mfn2 knock-out mice gain less weight when fed with a HFD (Dietrich et al., 2013). Interestingly, these changes in fusion-like mitochondrial dynamics are neuron-type specific because POMC-specific Mfn2 knockout mice exhibit hyperphagia, an increase in body weight and a decrease in energy expenditure (Schneeberger et al., 2013). In this mouse model, it is noteworthy that the number of mitochondria-ER contacts was significantly reduced in POMC neurons. This result indicates a synergy of action between hypothalamic RE and mitochondria in metabolic control. The change in mitochondrial dynamics in hypothalamic neurons directly affects distal metabolic organs and suggests a key role of mROS.

Other key molecular actors of mitochondrial function are uncoupling proteins (UCP) which are mitochondrial inner membrane proteins promoting proton leak (Aquila et al., 1985). These proteins control mROS release and specifically UCP2 which was described as a negative regulator of mitochondrial hydrogen peroxide production (Negre-Salvayre et al., 1997). Besides allowing protons to transit from the matrix to the inter-membrane space of the mitochondria, it has been suggested that UCP2 allows the transport of metabolites such as aspartate, malate and oxaloacetate. As such, UCP2 could reduce substrates of Krebs cycle, decrease electrons transport chain activity and also attenuates ROS production (Vozza et al., 2014). UCP2 is highly expressed in the hypothalamus (Richard et al., 1998), and more precisely in both POMC and NPY/AgRP neurons. This mitochondrial protein is strongly linked to nutritional status because the hypothalamic mRNA and protein expression of UCP2 is increased during the fasted state (Coppola et al., 2007). A hypothesis developed by Andrews et al., suggests that during negative energy balance (such as fasting or before a meal), NPY/AgRP neurons are activated via a decrease in basal mROS production, probably because of the activation of UCP2 (Andrews et al., 2008). Conversely, mROS levels in POMC neurons are also reduced, but these neurons are silent. In contrast, during positive energy balance, mROS are able to accumulate in POMC neurons to inhibit food intake. As expected, genetic inactivation of UCP2 attenuates the refeeding signal, due to an uncontrolled increase of ROS in NPY/AgRP neurons that lead to decrease their activities. These results underscore the importance of the level of ROS released, depending on the type of neurons.

As previously described, changes in glucose levels are sensed in the brain by GE and GI neurons. Arcuate nucleus POMC neurons are considered as GE while AgRP/NPY neurons are GI. Parton et al., have demonstrated the essential role of glucose sensing by POMC neurons in the overall physiological control of blood glucose (Parton et al., 2007). Specific genetic deletion of Kir6.2 subunit in K_ATP_ channels, required for glucose detection in POMC neurons, impairs the whole-body response to a systemic glucose load. In the same study, the authors demonstrated that UCP2 negatively regulates glucose sensing in POMC neurons. By using genipin (an UCP2 inhibitor) or UCP2 knock-out mice, they have induced an increase in POMC neurons firing rate. Glucose sensing by POMC neurons became defective in obese mice fed a HFD, suggesting that loss of glucose sensing has a causal role in the development of type 2 diabetes.

Thus, it appears that NPY/AgRP neurons activation is mediated by a decrease in ROS levels while POMC neurons activation is driven by ROS (Andrews et al., 2008). Indeed, icv administration of ROS scavengers induces significantly lower c-Fos expression in POMC neurons and increases food intake during light cycle, observed via an increase of c-Fos expression in AgRP/NPY neurons (Diano et al., 2011). Similarly, addition of H_2_O_2_ depolarizes POMC neurons, increases the firing rate, and an icv injection of H_2_O_2_ causes significantly less feeding of mice after an overnight fast.

**Figure 1:**
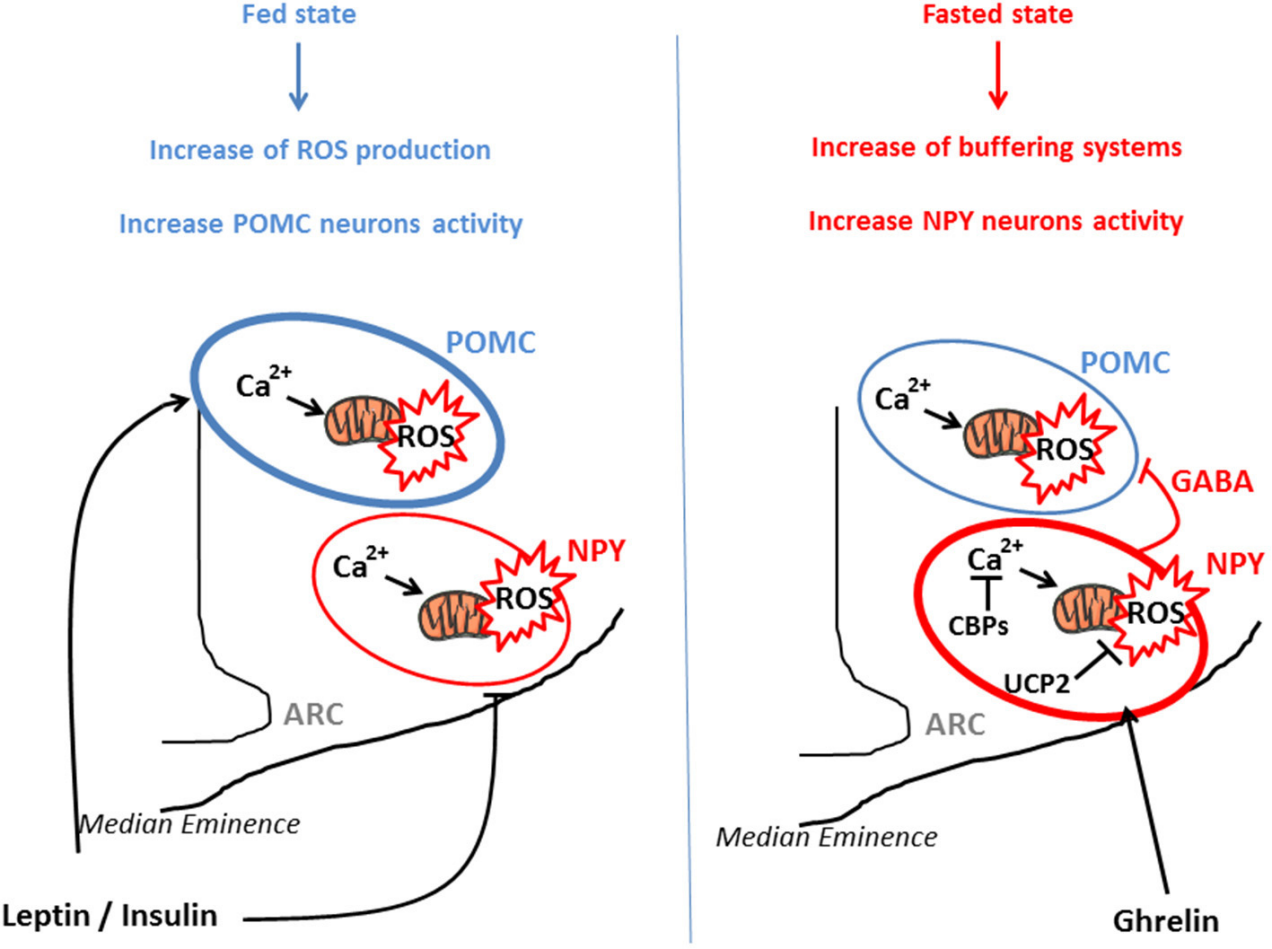
**POMC and NPY/AgRP activation via ROS release in function of nutritional state**. POMC and NPY/AgRP neurons generate opposing effects on food intake and metabolism. In the fed state, glucose, lipids, insulin, and leptin actions lead to ROS release in both type of neurons, for instance via a rise of Ca^2+^ concentration and mitochondrial dynamics. This transient increase in intracellular ROS stimulates POMC neurons activity and inhibits NPY/AgRP neurons, engendering decrease in food intake and increased energy expenditure. In the fasted state, NPY/AgRP neurons become more active, via an increase in Calcium Binding Proteins (CBPs) and UCP2 expression that respectively buffer excessive intracellular Ca^2+^ and prevent ROS release. This NPY/Agrp activation is mediated via ghrelin and stimulates food intake. In parallel, these neurons present GABAergic inhibitory synapses around POMC neurons, amplifying the orexigenic signal.

From a molecular perspective, POMC neurons seem to use glucose as their main fuel since their firing rate vary as a function of in glucose concentrations (Ibrahim et al., 2003). On the other hand, NPY/AgRP neurons are inhibited by high levels of glucose and may use free fatty acids as their main fuel (Mountjoy et al., 2007; Andrews et al., 2008). This differential fuel utilization underlies the presence of two distinct and competitive mechanisms in these neuronal populations: glycolysis and β-oxidation. When glycolysis is elevated, end-product derived from this reaction inhibit β-oxidation, and conversely, when β-oxidation is increased, glycolysis is inhibited. Both systems are opposite and produce ROS. An increase in the activity of NPY/AgRP neurons is followed by Ca^2+^ influx, which was demonstrated to elevate ROS production in neurons (Hernandez-Fonseca et al., 2008). However, NPY/AgRP neurons do not produce considerable amount of ROS, indicating that these neurons have cellular properties associated with low capacity for ROS production and/or high capacity to buffer ROS. These opposite effects of ROS release in POMC and NPY/AgRP neurons, that contribute to nutrient sensing and appetite regulation, have been demonstrated by the work of Kuo et al., By using an appetite-suppressing sympathomimetic agent, phenylpropranolamine (PPA), authors have shown the involvement of Protein Kinase C (PKC) in the generation of hypothalamic ROS which stimulates POMC neurons activity and inhibits NPY neurons (Kuo et al., 2011). Central inhibition of ROS production by icv infusion of glutathione attenuates PPA anorectic effect together with the icv injection of PKC oligonucleotide antisense. In a similar study, the same authors demonstrated that hypothalamic NF-kB signaling is also involved in decrease of food intake by PPA treatment. This anorectic effect is mediated by the decrease of NPY expression and the rise of the expression of endogenous antioxidants such as SOD or gluthatione peroxidase, demonstrating the impact of ROS (Kuo et al., 2012). Another essential actor which participate to the regulation of ROS production is Ca^2+^. Voltage-gated Ca^2+^ channels are expressed throughout the entire hypothalamus contributing to the increase of cytoplasmic Ca^2+^ levels in sensitive neurons (Marques-Da-Silva and Gutierrez-Merino, 2012). The maintenance of intracellular Ca^2+^ homeostasis depends on (1) the precise control of Ca^2+^-ATPases activity in the plasma membrane, (2) the Ca^2+^ uptake into intracellular compartments, (3) the influx through voltage-operated channels and 4) the effectiveness of Ca^2+^ buffering via Calcium Binding Proteins (CBPs) (Strehler et al., 2007; Nicholls, 2009). Excessive intracellular Ca^2+^ accumulation causes mitochondrial ROS production (Weber et al., 2010) and hypothalamic CBPs such as Secretagogin (Scgn) modulate this ROS release by buffering Ca^2+^(Gyengesi et al., 2012). Thus, Scgn which is widely expressed in the NPY/AgRP neurons (Gyengesi et al., 2013) from the ARC but not in POMC neurons, could explain the lower production of ROS by these neurons. There seems to be a direct requirement to buffer Ca^2+^ in NPY but not in POMC neurons, to maintain cellular firing rate. Thus, buffering Ca^2+^ controls ROS production and allows the modulation of neurons as a function of the nutritional state (Figure 1).

All these actors involved in transient ROS production in response to nutrients, clearly demonstrate a key role of ROS signaling as a real gauge of energy status, which induces a fine tuning control of energy metabolism and food intake.

### Hormones

Like nutrients, hormones which are key signals of the metabolic state of organs, must be detected by the hypothalamus to control energy homeostasis. We will now discuss the role of the orexigenic hormone ghrelin and of two anorectic hormones leptin and insulin. These hormones reach the hypothalamus, have their receptors on hypothalamic POMC and NPY/AgRP neurons and are clearly involved in the control of food intake and energy metabolism via signaling pathways involving ROS release.

#### Ghrelin

Ghrelin is a gut-derived hormone secreted into the bloodstream which activates NPY/AgRP neurons and increases food intake (Andrews, 2011). Levels of this hormone rise during the fasting state, making a signal of starvation for neurons. Ghrelin receptor (GHSR), a seven trans-membrane G-coupled protein linked to Gq, is predominantly expressed in NPY/AgRP neurons (Willesen et al., 1999). In response to intraperitoneal injection of ghrelin, the number of mitochondria in NPY neuronal perikarya is increased in a UCP2-dependent manner while POMC perikarya remains unaffected (Andrews et al., 2008). In the same way, ghrelin induces symmetric, putatively inhibitory synapses on POMC neurons correlated with recruitment of miniature inhibitory post-synaptic currents (mIPSCs) on these cells. Conversely, ghrelin significantly reduces the number of asymmetric, putatively excitatory synapses on POMC neurons. This synaptic plasticity demonstrates how ghrelin hyperpolarizes POMC neurons by activating inhibitory NPY/AgRP (especially GABAergic) inputs. In parallel, ghrelin increases UCP2 expression, which scavenges free radicals in NPY neurons without affecting ROS production in POMC neurons. All these effects previously described are linked to the decrease in ROS levels in NPY/AgRP via UCP2 since the effect of ghrelin is totally lost in UCP2 knock-out mice. The scavenging of ROS by icv infusion of a cocktail of antioxidants in UCP2 knock-out mice restores the action of ghrelin demonstrating the key role of hypothalamic ROS in ghrelin effects (Andrews et al., 2008).

From a molecular perspective, ghrelin activates AMPK in NPY neurons, which importantly raises mitochondrial respiration and ROS production (Andrews et al., 2008). Via the activation of GHSR on NPY neurons, ghrelin generates an increase in intracellular Ca^2+^ levels. Indeed, ghrelin induces a presynaptic signaling pathway that induces direct Ca^2+^ release from internal stores in NPY/AGRP neurons (Yang et al., 2011) and also activates Calmodulin-dependent protein Kinase Kinase (CaMKK) (Cummings et al., 2005). Activated CaMKK induces AMPK activation which generates ATP and fatty acid oxidation that might contribute to the neuron-specific activation of fatty acid oxidation and the resulting buffering of ROS (Horvath et al., 2009). CaMKK is particularly involved in the hypothalamic control of appetite because its deletion in NPY/AgRP neurons reduces food intake and body weight, while deleting CaMKK in POMC neurons does not affect energy homeostasis (Anderson et al., 2008; Gyengesi et al., 2012). Systemic injection of ghrelin fails to induce food intake in CamKK knock-out mice, demonstrating that CaMKK is also involved in the effect of ghrelin (Anderson et al., 2008). This synergy of effects between ROS and Ca^2+^ is necessary to generate signaling pathways in NPY/AgRP neurons which is involved in the action of ghrelin on food intake. Precise mechanisms need to be determined.

Finally, it is also known that motivation is an essential part of food intake, involving reward system and memory, which are regulated by dopamine neurons. Ghrelin has been shown to play a role for incentive value of food cue by controlling the activity of dopamine neurons (Abizaid et al., 2006; Andrews et al., 2006). Similar to its effect on NPY/AgRP neurons, ghrelin activates dopamine neurons in an UCP2-dependent manner and via a decrease of ROS production. Indeed, UCP2 knock-out mice exhibit a reduced dopamine turnover (Andrews et al., 2006). Besides its direct effect on the dopamine reward system, ghrelin regulates dopamine neurons directly via activation of AgRP neurons and inhibition of PVN, which induces food seeking behavior (Krashes et al., 2011; Atasoy et al., 2012).

#### Leptin

Leptin is an adipose tissue derived hormone (i.e., adipokine) which plays a central role in the regulation of energy homeostasis. Leptin acts on both NPY/AgRP and POMC neurons through its receptor LepRb (Morton and Schwartz, 2011). Leptin reaches hypothalamus and increases the activity of POMC neurons. This effect on POMC neurons induces a decrease in food intake and a rise in energy expenditure via the action of sympathetic nervous system on Brown Adipose Tissue (BAT) and muscles. Unlike ghrelin which modulates ROS levels in NPY/AgRP neurons, leptin seems to induce ROS levels in POMC neurons (Diano et al., 2011). Indeed, by using dihydroethidium (DHE; a substrate for fluorimetric detection of peroxidase as a readout of ROS), Diano et al., have demonstrated the lowest level of ROS in POMC neurons of leptin knock-out mice (ob/ob). In contrast, a 48 h leptin treatment in ob/ob mice results in elevated DHE expression in POMC neurons. This increase in DHE expression is also found in POMC neurons of fed wild-type mice compared with fasted mice, demonstrating a key role of ROS signaling in POMC neurons on the nutritional state. Moreover, the level of ROS in POMC neurons is correlated with those of leptin in wild-type and ob/ob mice, but this correlation is lost in HFD-fed mice. In parallel, the authors noted the presence of peroxisomes in POMC neurons (Diano et al., 2011). Indeed, in POMC neurons from ob/ob mice, the number of mitochondria and peroxisomes is very low while it is increased in lean mice. More particularly, mitochondria are outnumbered in lean fed mice compared with lean fasted mice, without differences in peroxisomes number. On the other hand, peroxisomes were almost three-fold higher in POMC neurons of HFD fed mice, suggesting a role of this organelle in leptin resistance and specifically in the disruption of ROS release in POMC neurons. Hydrogen peroxide icv injection in HFD-fed mice resulted in increased c-Fos expression in POMC neurons and decreased feeding in response to peripheral leptin injection. This spectacular experiment has proved that ROS alone could reverse POMC function and mediate leptin actions.

Leptin was also shown to be a key molecular actor involved in synaptic plasticity around POMC neurons (Pinto et al., 2004). In ob/ob mice, a significantly greater number of excitatory synapses (EPSCs) on NPY neurons was observed, while the number of inhibitory synapses (IPSCs) is higher on POMC neurons. After 2 days of intraperitoneal injection of leptin, NPY neurons display an 85% reduction of the number of EPSCs and an almost 70% increase in the number of IPSCs. At the same time, there is also a doubling of the synapses number on POMC neurons, with an almost 300% increase in the number of EPSCs. This regulation resembles those of ghrelin on synaptic plasticity (Andrews et al., 2008), suggesting again an involvement of ROS. Similarly, the effect of leptin are potentiated by intracellular Ca^2+^ like ghrelin (Qiu et al., 2010).

#### Insulin

In addition to its peripheral effect on glucose storage, insulin acts on the hypothalamus to modulate energy metabolism and food intake (Abraham et al., 2014). In particular, insulin inhibits food intake in the hypothalamus through ROS production. A first study has demonstrated the key role of the mitochondrial respiratory chain in the phosphorylation of insulin receptor on neuronal cells (Storozhevykh et al., 2007). This study suggests that insulin might trigger ROS production within the hypothalamus, which, in turn, inhibit food intake. Indeed, insulin injection in the third ventricle generates a transient ROS release 15 min post-injection and induces food intake (Jaillard et al., 2009). This insulin-induced ROS increase is transient because no ROS are observed at either 5 or 30 min post-injection. Moreover, this insulin-induced ROS increase is dependent on the nutritional state, because a decrease of food intake is not observed in fasted mice. The antioxidant trolox delivery by icv injection 30 min prior to insulin completely prevents insulin-stimulated ROS increase and suppresses insulin-induced food intake inhibition. Interestingly, the mROS seems to be not involved in this insulin effect. Indeed, icv injection of an inhibitor of NADPH oxidase (DPI) 30 min before insulin treatment inhibits the effect of insulin on ROS production and food intake.

## Consequences of the alteration of hypothalamic ROS release in pathological state

The short-term hypothalamic ROS peak generated by metabolic signals (nutrients and hormones) appears to be fundamental to elicit a proper behavioral, endocrine and autonomic response to nutrient intake. A pathology characterized by a disruption in nutrient sensing and by an inability to maintain energy homeostasis is type 2 diabetes (T2D) (Schwartz and Porte, 2005). T2D is especially characterized by impaired metabolic and hormonal hypothalamic sensing, and by a chronic whole-body low-grade inflammatory background (Purkayastha and Cai, 2013). An important part of this low-grade inflammation is due to oxidative stress. As previously described, transient ROS release is now considered as a molecular signal. When ROS production is chronically elevated, the over-abundance of radical species triggers activation of inflammatory kinases, leading to alterations of tissue functions. This phenomenon is exacerbated by a decrease in the expression of antioxidant enzymes (Gonzalez-Chavez et al., 2011). In particular in the brain, this pathological ROS release induces over-activation of the sympathetic nervous system (Hirooka et al., 2011). This rise of sympathetic excitation is responsible for disorders associated with obesity and T2D such as hypertension, cardiovascular and renal dysfunctions (Campese et al., 2004), and antioxidant icv injection improves this deleterious phenotype. Oxidative stress and inflammation are also develop in the hypothalamus during T2D (Purkayastha and Cai, 2013).

In this review, transgenic models of obesity or nutritional models (HFD) mice will be considered as type 2 diabetic mice.

### Oxidative stress in the hypothalamus during T2D

The different characteristics of T2D suggest an alteration of redox signaling and ROS release in the hypothalamus. Zücker rats are obese and insulin resistant with dramatic autonomic disturbances including modification of sympathovagal balance (York et al., 1985). In this model, a hypothalamic hypersensitivity to glucose has been demonstrated (Colombani et al., 2009). In lean rats, intracarotid injection of low-dose of glucose (3 mg/kg) does not generate ROS release nor a peak of insulin secretion, while a most important dose of glucose (9 mg/kg) induces insulin release 1–3 min later associated with hypothalamic mROS production. This same low-dose of glucose (3 mg/kg) is sufficient to produce a rapid and transient increase in plasma insulin in Zücker rats. Similarly, low-dose of glucose increases hypothalamic ROS in Zücker rats. These diabetic rats present an altered redox state, together with an increase of hypothalamic mitochondrial activity in response to substrates, and a decrease in antioxidant enzymes activity. In rats, icv infusion of glutathione for 3 days restores a normal hypothalamic glucose sensing.

In association with these data, our group has observed that ROS, and more particularly H_2_O_2_ release, is exacerbated in hypothalamus of HFD-fed mice compared with normal mice (Duparc et al., 2011a; Drougard et al., 2014). In this study, we demonstrated that icv injection of high levels of apelin, similar to that observed in the hypothalamus of obese/diabetic mice (Reaux-Le Goazigo et al., 2011), in normal mice, induces a diabetic phenotype with fasting hyperglycemia, fasting hyperinsulinemia and glucose intolerance. In fact, these disturbance of glucose homeostasis via hypothalamic apelin are due to an over-activation of sympathetic nervous system that triggers hepatic glucose production. Central apelin effects are directly linked to hypothalamic over-production of H_2_O_2_, since an icv injection of trolox 30 min before apelin icv injection inhibits the establishment of T2D characteristics. Production of hypothalamic H_2_O_2_ seems to have a mitochondrial origin, since icv injection of DPI 30 min before apelin treatment does not inhibit fasting hyperglycemia. This high level of hypothalamic H_2_O_2_ release reflects an oxidative stress. Indeed, this release is exacerbated in hypothalamus of HFD-fed mice in response to apelin, and is inhibited by trolox pretreatment. Moreover, over-expression of apelin by lentivectors in the VMH promotes an increase of pro-inflammatory cytokines expression (Drougard et al., 2014), which are known to alter the control of metabolism (De Souza et al., 2005).

### Alterations of ROS signaling in POMC and NPY/AgRP neurons during T2D

#### POMC neurons

Neuronal alterations observed in genetic diabetic mice in response to glucose are also found in HFD-fed mice (Parton et al., 2007). Approximately 46% of POMC neurons from lean mice significantly increase their firing rate after a rise in glucose concentration from 3 to 5 mM compared with HFD-fed mice, in which only 10% of POMC neurons respond. Moreover, glucose fails to enhance the release of α-MSH in POMC neurons of HFD-fed mice. We have previously described that POMC neurons need to release ROS to be effective in response to glucose, and UCP2 inhibits ROS production. As expected, UCP2 expression is increased in hypothalamus of HFD-fed mice and genipin treatment restores α-MSH release in response to glucose in POMC neurons of HFD-fed mice. These results are confirmed by the protection from obesity, T2D and alteration of glucose sensing in POMC neurons from UCP2 knock-out mice.

An increase in ROS production in POMC neurons compared to NPY neurons under “basal” conditions has been observed, suggesting that POMC neurons might be prone to free radical-induced degeneration (Andrews et al., 2008). The decline in functional POMC neurons may promote increased orexigenic NPY tone, hyperphagia and weight gain. Indeed, ROS levels are significantly higher in POMC neurons of HFD-fed mice compared to chow-fed mice (Diano et al., 2011). The lack of difference in ROS levels in POMC neurons between chow-fed mice and HFD-fed mice is associated with the loss of correlation between leptin and ROS levels in HFD-fed mice. Indeed, the absence of a positive correlation between elevated leptin levels and ROS release in HFD-fed mice, could be explained by the important rise of peroxisomes in POMC neurons of these mice. This proliferation of peroxisome is governed by the nuclear receptor peroxisome proliferator-activated receptor-γ (PPAR-γ) (Green, 1995) which is associated with brain inflammation, gliosis (Bernardo and Minghetti, 2006) and ROS production (Yu et al., 2008). PPAR-γ expression is increased in hypothalamic POMC and NPY/AgRP neurons of HFD-fed mice (Diano et al., 2011). These peroxisome in POMC neurons interfere clearly with ROS signaling, since icv treatment with a PPAR-γ agonist in normal mice elevates peroxisome number, decreases ROS production and increases food intake. Conversely, icv treatment with a PPAR-γ antagonist in HFD-fed mice decreases peroxisome number, increases ROS in POMC neurons and reduces daily food intake. Consistent with these results, icv treatment with a PPAR-γ antagonist in HFD-fed mice increases c-Fos staining and firing rate in POMC neurons, and decreases NPY/AgRP neurons excitation compared with HFD control mice. As expected, PPAR-γ knock-out mice fed with a HFD are protected from obesity and T2D (Long et al., 2014). These mice present a decrease in peroxisomes density in POMC neurons, which allows ROS signaling and firing rate in these neurons. On a metabolic standpoint, these mice are protected from hyperphagia, body weight gain, decrease of energy expenditure and leptin resistance. As previously described, this ROS signaling is overriding in POMC neurons, and icv injection of H_2_O_2_ in HFD-fed mice induces c-Fos expression in POMC neurons, decreases feeding and improves leptin sensitivity in response to peripheral leptin injection (Diano et al., 2011). It has been shown that only 3% of POMC neurons from HFD-fed mice are sensitive to leptin, and that H_2_O_2_ treatment leads to an 18% increase of POMC neurons, demonstrating the key role of ROS signaling from POMC neurons for leptin sensitivity.

#### NPY/AgRP neurons

NPY/AgRP neurons also seem affected, because if ROS generation is uncontrolled in these cells, neuronal firing is impaired. To this aim, emerging concept indicates that T2D is also characterized by ghrelin resistance, since ghrelin fails to induce AgRP or NPY secretion in hypothalamic explants from HFD-fed mice (Andrews et al., 2008; Briggs et al., 2010). This global hypothalamic oxidative stress disrupts all the redox balance in POMC, but also in NPY/AgRP neurons, which alters nutrient sensing and energy balance. Indeed, insulin, an anorectic hormone, fails to induce a decrease of food intake in HFD-fed mice, demonstrating a lack of responsiveness of hypothalamus to peripheral signals (Storozhevykh et al., 2007).

#### Mitochondrial dynamics

From the viewpoint of the mitochondrial dynamics, VMH mitochondrial fission via DRP1 is essential to respond to glucose load (Carneiro et al., 2012). Moreover, mitochondrial density in POMC neurons from HFD-fed mice was increased (Diano et al., 2011). These mitochondria present a significant reduction in the number of contacts with ER, indicating that both mitochondrial network complexity and their association to ER in POMC neurons are altered in HFD-fed mice (Schneeberger et al., 2013). The protein involved in mitochondrial fusion protein Mfn2 seems to be the most crucial protein implicated in the HFD-fed mice phenotype, since its hypothalamic expression is decreased in HFD-fed mice when compared to other mitochondrial fusion/fission proteins such as DRP1 and Mfn1. Indeed, Mfn2 over-expression in ARC neurons decreases food intake and body weight, while Mfn2 knock-out mice demonstrate an increase in food intake and body weight, a decrease of energy expenditure and a resistance to leptin. These mice also present an alteration of the mitochondrial respiratory capacity and an increase in ROS production. This over-production of ROS appears to be a consequence of hypothalamic ER stress. In fact, injection of ROS scavenger does not improve the phenotype of these mice, whereas inhibition of ER stress restores a normal phenotype.

### Treatments against type 2 diabetes in connection with the hypothalamic ROS

T2D is a worldwide expanding disease that is difficult to treat because of multiple organ dysfunction. So far, therapies have targeted insulin-sensitive organs such as muscles, liver and adipose tissue to improve hyperglycemia, hyperinsulinemia and resistance to insulin (Altaf et al., 2014). However, considerable progress that have been made in the field of neurophysiology have demonstrated a key role of the brain, and more particularly the hypothalamus, in the control of energy homeostasis. It is now established that ROS release in hypothalamic neurons is necessary to generate the “periphery-hypothalamus-periphery” regulation loop in response to the nutritional state. This hypothalamic ROS balance finely regulated could be altered and generate oxidative stress, which is a disruption of the balance between oxidant and antioxidant molecules in favor of oxidant molecules, masking beneficial effects of ROS (Sies and Cadenas, 1985). The best treatment against T2D is calorie restriction (Bantle, 1988) to avoid abundance of substrates for mitochondria, and reduce the ROS over-production. This change in lifestyle is efficient in humans in the prediabetic state, however patient's adherence is poor. Regarding direct targeting of oxidative stress, treatments with antioxidants could have the potential to prevent or delay the development of diabetic complications. Despite the preponderance of evidence from animal studies supporting this hypothesis, large scale prospective studies using antioxidant treatments in humans have been disappointingly inconclusive, probably because of issues with dosage and bioavailability of these molecules (Johansen et al., 2005; Pazdro and Burgess, 2010).

### Oxidative stress, a link between T2D and others diseases

Over-production of ROS (arising either from mitochondrial electron-transport chain or excessive stimulation of NADPH oxidase) results in oxidative stress, a deleterious process that can be an important mediator of cell damage. Indeed, hypothalamic oxidative stress during T2D is a risk factor for many chronic diseases such as neurodegenerative disorders (Valko et al., 2007). It has been clearly demonstrated that T2D increases the risk of the development of Alzheimer and Parkinson diseases via brain oxidative stress (Butterfield et al., 2014; Santiago and Potashkin, 2014). It is particularly interesting to underlie that both neuronal pathologies are characterized by mitochondrial dysfunctions inducing alteration of brain ROS release.

Given the failure of care therapies against T2D coupled to the massive expansion of the disease and the associated-pathologies; it is critical to elaborate integrated therapeutics. Indeed, we have shown in this review the key role of ROS production in hypothalamus with a different pattern of release between POMC and NPY/AgRP neurons. ROS signaling is largely altered in T2D, and the hypothalamus has an “open-access” to bloodstream signals via the median eminence. Via this anatomical characteristic, it is now primordial to evaluate the consequences of new therapies on this hypothalamic balance sensing. Therefore, it will be interesting to elaborate non-invasive treatments to normalize this hypothalamic ROS production via targeting all molecular actors involved in this ROS release control. To this aim, a study of our group has demonstrated the alteration of hypothalamic NO release via the dysfunction of gut-brain axis in response to glucose in db/db mice (Duparc et al., 2011b). Modulation of this gut-brain axis could be an attractive therapeutic strategy to normalize hypothalamic ROS production.

## Conclusion

Energy metabolism and food intake are complex processes that involve homeostatic mechanisms driven by hypothalamic network signaling pathways. This review brings light to the fact that hypothalamic ROS levels, controlled by various molecules and fuel signals, play an important role in this hypothalamic control of energy balance. The control of hypothalamic ROS release involves many molecular actors such as mitochondrial dynamics, NADPH oxidase activity, synaptic plasticity, peroxisomes, endoplasmic reticulum,… At high levels that saturate buffering mechanisms, ROS may initiate cellular degeneration in either NPY/AgRP or POMC neurons, and impair energy homeostasis, resulting in pathologies such as obesity and type 2 diabetes. In addition to the brain, future therapeutic strategies have to take into account the large part played by ROS signaling in other structures (including pancreas, adipose tissue, intestine, liver and heart).

### Conflict of interest statement

The authors declare that the research was conducted in the absence of any commercial or financial relationships that could be construed as a potential conflict of interest.
